# And yet, it moves: nuclear and chromatin dynamics of a heterochromatic double-strand break

**DOI:** 10.1098/rstb.2016.0291

**Published:** 2017-08-28

**Authors:** P. Christopher Caridi, Laetitia Delabaere, Grzegorz Zapotoczny, Irene Chiolo

**Affiliations:** Department of Molecular and Computational Biology, University of Southern California, Los Angeles, CA 90089, USA

**Keywords:** heterochromatin repair, homologous recombination, genome stability, nuclear architecture, repeated DNA sequences, *Drosophila*

## Abstract

Heterochromatin is mostly composed of repeated DNA sequences prone to aberrant recombination. How cells maintain the stability of these sequences during double-strand break (DSB) repair has been a long-standing mystery. Studies in *Drosophila* cells revealed that faithful homologous recombination repair of heterochromatic DSBs relies on the striking relocalization of repair sites to the nuclear periphery before Rad51 recruitment and repair progression. Here, we summarize our current understanding of this response, including the molecular mechanisms involved, and conserved pathways in mammalian cells. We will highlight important similarities with pathways identified in budding yeast for repair of other types of repeated sequences, including rDNA and short telomeres. We will also discuss the emerging role of chromatin composition and regulation in heterochromatin repair progression. Together, these discoveries challenged previous assumptions that repair sites are substantially static in multicellular eukaryotes, that heterochromatin is largely inert in the presence of DSBs, and that silencing and compaction in this domain are obstacles to repair.

This article is part of the themed issue ‘Chromatin modifiers and remodellers in DNA repair and signalling’.

## 1. Heterochromatin presents unique challenges to DNA repair

DNA is under constant attack from both endogenous and exogenous stresses, resulting in various lesions to the double helix. Double-strand breaks (DSBs) are the most dangerous type of DNA damage, because they interrupt the continuity of the DNA molecule: even a single DSB can trigger cell death or genomic instability if left unrepaired [1–5]. Importantly, DSB repair occurs in the context of chromatin, which comprises histones and non-histone proteins that package the DNA and influence several aspects of DNA damage processing and repair (reviewed in [6,7]).

Two main types of chromatin have been described in eukaryotic cells: euchromatin and heterochromatin. Heterochromatin (from the Greek words ‘heteros’ = different, and ‘chroma’ = colour) was initially defined based on distinctive histological staining patterns in interphase cells, where it appeared as more densely stained regions of the nucleus amid lightly stained euchromatin [8]. We now know that these two types of chromatin represent two distinct genomic and nuclear domains distinguished by several properties, including histone modifications, chromatin accessibility, gene density, replication timing and DNA sequence composition ([9,10]; reviewed in [11]). While much is known about DSB repair pathways in euchromatin, heterochromatin repair mechanisms are just starting to emerge.

**Figure 1.**
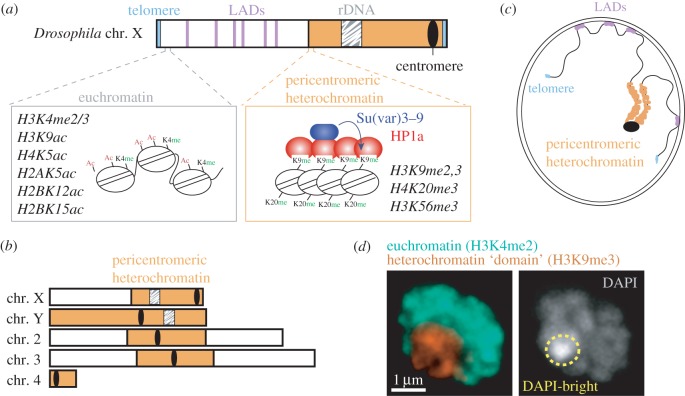
Organization and distribution of heterochromatin in *Drosophila*. (*a*) Organization of different types of silenced sequences along a *Drosophila* chromosome, including distinguishing features between euchromatin and pericentromeric heterochromatin in terms of chromatin compaction and histone modifications. HP1a and Su(var)3–9 are enriched in heterochromatin (the arrow indicates that Su(var)3–9 maintains and spreads H3K9me2/3 in heterochromatin). (*b*) Schematic view of all *Drosophila* chromosomes showing the position and extent of pericentromeric heterochromatin (adapted from [9]). (*c*) Schematic view of the nuclear position of different types of silenced sequences relative to the nuclear periphery in *Drosophila* cells. (*d*) Immunofluorescence analysis of a *Drosophila* Kc cell (adapted from [13]), showing the organization of heterochromatin in a distinct nuclear domain, surrounded by euchromatin. The DAPI-bright region is embedded in the heterochromatin domain.

Heterochromatin is typically enriched for the ‘silent’ histone marks H3K9me2/3, and associated proteins like heterochromatin protein 1a (HP1a) in flies [9,12] (figure 1*a*) and HP1α or HP1β in mammalian cells [14]. Conversely, histone modifications correlated with ‘open’ chromatin and gene expression (e.g. histone hyperacetylation and H3K4me) are generally found in gene-rich, euchromatic regions [15–18] (figure 1*a*,*b*). Heterochromatin is also more compact than euchromatin, resulting in reduced accessibility to molecules and enzymatic digestion [19–22]. In terms of chromosomal localization, most heterochromatin is concentrated at pericentromeric and telomeric regions in *Drosophila* and mammalian cells, while euchromatin is distributed along the chromosome arms (figure 1*a*) [9,10,15–18]. This review focuses on pericentromeric heterochromatin, a prominent chromosomal structure spanning about 30% of fly and human genomes [10,23,24] (figure 1*b*), but absent in budding yeast. Notably, pericentromeric heterochromatin is late replicating in most organisms [25,26], but it is functionally and structurally distinct from late replicating lamina-associated domains (LADs) distributed along the chromosome arms [10,27–29]. In contrast to those, pericentromeric heterochromatin is not usually associated with the nuclear periphery (e.g. [13,30–35]) or enriched for H3K27me3 which promotes tissue-specific gene silencing (also traditionally referred to as ‘facultative heterochromatin’) (figure 1*c*) [10].

Pericentromeric heterochromatin (hereafter ‘heterochromatin’) is mostly composed of repeated DNA sequences [10,23,24]. In *Drosophila*, for example, about half of these sequences are ‘satellite’ repeats, predominantly 5 base-pair sequences repeated in tandem for hundreds of kilobases to megabases, while the rest of the heterochromatin contains scrambled clusters of transposable elements and about 250 isolated genes [10,23,24]. Heterochromatin is likely maintained in cells because of its critical roles in centromere function [36–38], sister chromatid cohesion [39,40], meiotic pairing [41,42] and genome organization [35,43], but the abundance of repeated sequences also presents unique challenges to DSB repair and genome stability (reviewed in [31,44,45]).

The two prominent repair pathways responding to DSBs are non-homologous end joining (NHEJ) and homologous recombination (HR). NHEJ involves direct re-joining of the two ends with little processing and is frequently error-prone [46–50] (reviewed in [51]). Conversely, HR relies on extensive resection of the DSB to form single-stranded DNA (ssDNA) filaments, which invade ‘donor’ homologous sequences used as templates for DNA synthesis and repair (reviewed in [52]). In single copy sequences, a unique donor is present on the sister chromatid or the homologous chromosome, and HR repair is largely ‘error free’ [52]. In heterochromatin, however, the availability of thousands to millions of potential donor sequences in pericentromeric regions of different chromosomes can initiate unequal sister chromatid exchanges, or intra-/inter-chromosomal recombination, leading to deletions, duplications, translocations, release of DNA circles, and formation of dicentric or acentric chromosomes [13,32,53–55] (reviewed in [31,44,45]). Despite this risk, HR is a primary pathway used to repair heterochromatic DSBs in both *Drosophila* and mammalian cells [13,32,34,55–57], and specialized mechanisms enable ‘safe’ HR repair in heterochromatin while preventing aberrant recombination.

**Figure 2.**
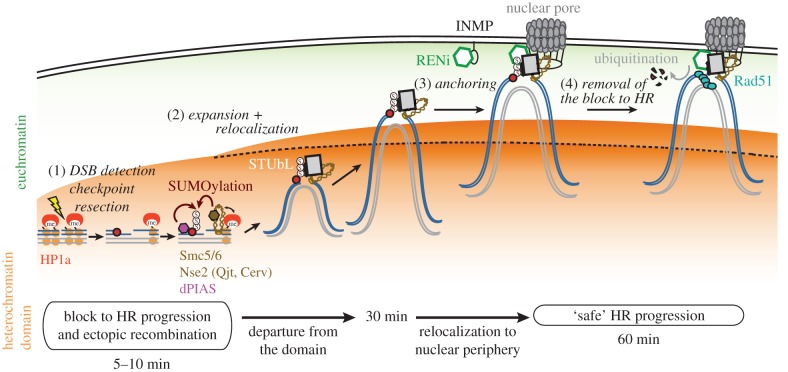
Model for the molecular mechanisms that relocalize heterochromatic DSBs to the nuclear periphery in *Drosophila*. When DSBs form in heterochromatin (orange area), early damage responses efficiently occur inside the domain. These include DSB detection, checkpoint activation, resection, and the recruitment of Smc5/6 (including its SUMO-E3 ligase subunits Nse2/Qjt, Nse2/Cerv) and the SUMO-E3 ligase dPIAS. SUMOylation of unknown targets blocks HR progression inside the heterochromatin domain, thus preventing ectopic recombination. SUMOylated proteins recruit the STUbL protein Dgrn, and induce relocalization of repair sites to nuclear pores (as shown) or INMPs, at the nuclear periphery. The RENi protein dRad60 associates with STUbL and Smc5/6, at the nuclear periphery. Anchoring to the nuclear periphery promotes STUbL-mediated ubiquitination of SUMOylated targets, removal of the block to HR progression, Rad51 recruitment, and ‘safe’ HR progression. Removal of the block might rely on proteasome-mediated degradation of ubiquitinated targets (as shown). Alternatively, these targets might become active after ubiquitination or de-SUMOylation (not shown). This model also predicts that sister chromatids or homologous chromosomes (grey lines) relocalize in concert with the damaged site to provide homologous templates for repair completion. min: time in minutes after DSB formation by exposure to ionizing radiation.

**Figure 3.**
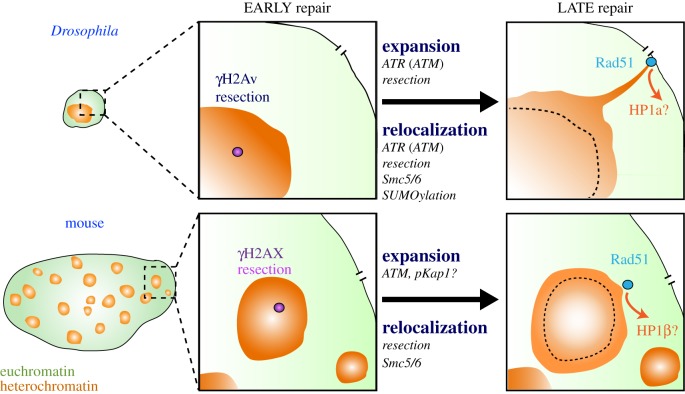
Heterochromatic double-strand break (DSB) repair in *Drosophila* or mouse cells in S/G2. Heterochromatin (orange) is organized in one distinct domain in *Drosophila* (top left) and several chromocentres in mouse cells (bottom left). Cells are drawn to scale. In both systems, DSB repair starts with the phosphorylation of H2Av/H2AX and continues with resection inside the heterochromatin domain (purple foci). Next, repair sites relocalize to outside the domain and, at least in *Drosophila*, DSBs reach the nuclear periphery before recruiting Rad51 and continuing repair. Relocalization requires resection and occurs during heterochromatin expansion in both systems. Heterochromatin repair also relies on HP1β and Kap1 phosphorylation by ATM in mouse cells, likely resulting in chromatin loosening, Chd3 release and repair progression. Similarly, in fly cells HP1a displacement correlates with Rad51 recruitment, suggesting a local relaxation of the chromatin state during heterochromatin repair. Components and pathways indicated under the words ‘expansion’ and ‘relocalization’ refer to molecular mechanisms required for these processes.

Studies in *Drosophila* cells, where heterochromatin forms a distinct nuclear ‘domain’ [9,13] (figure 1*d*), revealed that HR starts inside the domain, leading to resection [13,32,57,58], but subsequent repair steps are temporarily halted [13,32,55] (figure 2). Next, resection triggers a global expansion of the domain and a striking relocalization of DSBs to the nuclear periphery, where repair progresses [13,32,57,58] (figures 2 and 3). Interestingly, ‘silent’ chromatin marks are necessary for this spatial and temporal regulation of heterochromatin repair [13]. Inactivating this relocalization pathway results in aberrant recombination and widespread genomic instability, revealing its importance to genome integrity [13,32,55]. Relocalization likely promotes ‘safe’ HR repair while preventing aberrant recombination by isolating the DSBs and their repair templates (on the homologous chromosome or the sister chromatid) away from non-allelic (ectopic) sequences before strand invasion [13,32,55] (reviewed in [31,45,59]). Remarkable similarities to this relocalization pathway have been described in mouse cells [31,34,60,61] (figure 3), where heterochromatin is organized in several nuclear domains called ‘chromocentres’ [62], suggesting highly conserved strategies for heterochromatin repair [45].

This review will summarize our current understanding of the molecular mechanisms of heterochromatin repair in *Drosophila* and mouse cells, with a specific focus on the role of nuclear architecture and chromatin structure in different steps of repair. We will also highlight important similarities with pathways first described in yeast for DSB repair of other types of repeated sequences and persistent DSBs, which raise interesting questions regarding the nature of the signals responsible for relocalization.

## 2. Despite the risk of aberrant recombination, double-strand breaks in heterochromatin are efficiently processed for homologous recombination repair

**Figure 4.**
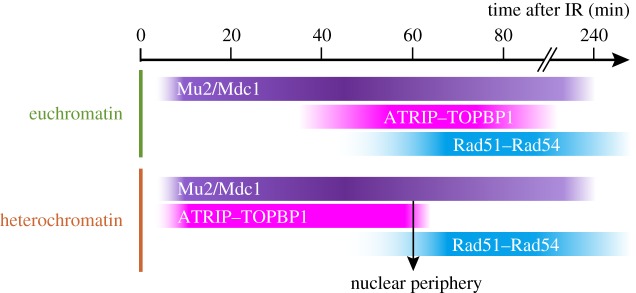
Early HR steps are enhanced in heterochromatin. Comparison of times of focus formation and disappearance of repair components associated with DSB detection (Mu2/Mdc1), resection (ATRIP and TopBP1) and strand invasion (Rad51 and Rad54) reveals different kinetics of early repair steps in euchromatin and heterochromatin in response to IR [13]. In heterochromatin, the downward arrow at 60 min indicates the association of repair sites to the nuclear periphery, which results in rapid ATRIP displacement and Rad51 recruitment [32].

Initial studies in mouse and *Drosophila* cells revealed that, in spite of the silent and compact nature of heterochromatin, DSB detection and signalling are not delayed in this domain [13,31,61]. In fly cells, responses associated with initial repair steps, including the formation of foci of γH2Av (corresponding to mammalian γH2Ax [63]) and Mu2/Mdc1 [13,64] (a component associated with γH2Av), occur within seconds to minutes from DSB induction with ionizing radiation (IR) [13], and with kinetics surprisingly similar to foci in euchromatin [13,57] (figure 4). This is in agreement with earlier studies showing that heterochromatin does not block access or exchanges of molecules [20–22].

Another unexpected feature of the DSB response in heterochromatin is that, despite the risk of aberrant recombination, HR is widely used for repairing this domain. *Drosophila* tissues enriched for G1 cells, and mammalian cells in G1/G0, largely use NHEJ in heterochromatin [30,34,56,57]. However, HR repair prevails in *Drosophila* cultured cells, which are mostly in S/G2 [13,32,55], as well as in mammalian cells during G2 [34,56,65], suggesting that HR is preferentially used in heterochromatin when both HR and NHEJ are available (reviewed in [45]). Perhaps even more surprising, foci of proteins that associate with resected DSBs (e.g. ATRIP and TopBP1) form faster and appear brighter in heterochromatin relative to euchromatin [13] (figure 4). This reveals that early steps of HR repair (e.g. resection, ATRIP/TopBP1 recruitment and/or focus clustering [31]) occur more efficiently in heterochromatin than in euchromatin [13].

Interestingly, resection is required for relocalizing heterochromatic DSBs to outside the domain in both *Drosophila* and mouse cells [13,34] (figure 3), revealing that this early response is important for the spatial and temporal dynamics of heterochromatin repair. Efficient resection in heterochromatin might represent an advantage because faster processing of a DSB facilitates its departure from the heterochromatin domain, thus preventing ectopic exchanges. At the same time, channelling DSBs towards the HR pathway provides more opportunities to regulate repair progression, given that this pathway is characterized by metastable intermediates that can be reverted in case of accidental strand invasion of ectopic sequences (reviewed in [52]).

Together, these studies reversed the initial assumption that heterochromatin is resistant to DSB processing and repair, and revealed that early HR steps are particularly efficient in this domain. However, studies in mouse and human cells also suggest that heterochromatin requires more time to complete repair than euchromatin [30,56], raising the possibility that repair is delayed at later stages in these cells.

## 3. Homologous recombination progression is halted inside the heterochromatin domain by SUMOylation to prevent aberrant recombination

While early steps of HR occur efficiently inside the heterochromatin domain in both *Drosophila* and mouse cells, recruitment of the strand invasion component Rad51 does not occur until after relocalization [13,32,34] (figures 2 and 3). In *Drosophila* cells, the initial block to HR progression is dependent on SUMOylation, with three SUMO E3 ligases involved: dPIAS and the Smc5/6 subunits Nse2/Qjt (Quijote) and Nse2/Cerv (Cervantes) [13,32,55] (figure 2 and table 1). SUMO E3 ligases are recruited to heterochromatic DSB before relocalization [13,32,55]. Removing these components results in abnormal recruitment of Rad51 inside the domain, aberrant recombination leading to heterochromatic DNA filaments between mitotically dividing cells, and widespread chromosome rearrangements [13,32,55]. These discoveries revealed the importance of SUMOylation and of the block to Rad51 recruitment inside the heterochromatin domain to prevent aberrant recombination between heterochromatic repeated sequences.

**Table 1.** Heterochromatin repair components. The main repair components responsible for heterochromatin repair in *Drosophila* are shown, including their functions in heterochromatic HR repair and homologous proteins in *S. cerevisiae* and mammalian cells. See text for details. Common names used in flies are in square brackets. Question marks indicate functions that have been hypothesized but not directly tested.

*D. melanogaster*	enzymatic/structural activity	function in *Drosophila* heterochromatic DSB repair	ref.	*S. cerevisiae*	mammals
Su(var)3–9, SetDB1 [Egg]	H3K9me2/3 methyltransferases	HP1a recruitment to heterochromatin.	[13]		Suv39H1 Suv39H2 SetDB1 SetDB2
HP1a [Su(var)205]	H3K9me2/3- associated protein	Smc5/6 recruitment to the heterochromatin domain. Maintains compaction.	[13]		HP1α HP1β HP1γ
ATR [Mei41] ATM [Tefu]	checkpoint kinases	Heterochromatin expansion. Relocalization of DSBs.	[13]	Mec1 Tel1	ATR ATM
Blm, Exo1 [Tosca], CtIP	resection proteins	Heterochromatin expansion. Relocalization of DSBs.	[13]	Sgs1, Exo1, Sae2	Blm, Exo1 CtIP
Smc5/6	core complex subunits of the Smc5/6 complex	Block HR progression and aberrant recombination inside the heterochromatin domain. Relocalization of DSBs.	[13]	Smc5/6	Smc5/6
Qjt, Cerv	SUMO-E3 ligase subunits of the Smc5/6 complex.	Block HR progression and aberrant recombination inside the heterochromatin domain. Relocalization of DSBs.	[32]	Mms21	Nse2
dPIAS [Su(var)2–10]	SUMO E3 ligase	Blocks HR progression and aberrant recombination inside the heterochromatin domain. Relocalization of DSBs.	[55]	Siz1, Siz2	PIAS1 PIAS2 PIAS3 PIAS4
Dgrn	SUMO-targeted Ub ligase (STUbL)	Relocalization/anchoring of DSBs. Repair restart.	[32]	Slx5/8	Rnf4
dRad60	SUMO-like protein associated with STUbL	Anchoring of DSBs. Repair restart?	[32]	Esc2	Nip45
Nup107	nuclear pore complex subunit	Anchoring of DSBs to the nuclear periphery. Repair restart.	[32]	Nup84	Nup107
Koi, Spag4	inner nuclear membrane proteins	Anchoring of DSBs to the nuclear periphery. Repair restart.	[32]	Mps3	Sun1, Sun2, Sun3, Sun5, Spag4

Interestingly, the loss of Smc5/6 leads to abnormal formation of Rad51 foci inside the heterochromatin domain in *Drosophila* [13,32,55], but not in mouse cells [34], suggesting the existence of alternative or redundant mechanisms that block HR progression in mammalian heterochromatin. Together, these studies uncovered pathways that halt HR progression inside the heterochromatin domain, and a central role of SUMOylation in this response in *Drosophila* cells.

## 4. Heterochromatic double-strand breaks relocalize to the nuclear periphery to continue homologous recombination repair

In *Drosophila* cells, heterochromatic DSBs associate with the nuclear periphery before recruiting Rad51 and continuing repair [13,32] (see also [45] for a recent review). Specifically, DSBs move to nuclear pores or to inner nuclear membrane proteins (INMPs) of the SUN family Koi and Spag4 [32] (figure 2 and table 1). At nuclear pores, this interaction is mediated by the ‘Y complex’ subunits Nup107-Nup160 [32] (table 1). Depletion by RNA interference (RNAi) of nuclear pores and INMPs results in increased dynamics of repair sites, persistent damage in heterochromatin and gross chromosomal rearrangements [32], revealing the importance of DSB anchoring to the nuclear periphery for accurate progression of heterochromatin repair.

Importantly, SUMO and the SUMO E3 ligases dPIAS, Nse2/Qjt and Nse2/Cerv, are required to relocalize DSBs to the nuclear periphery [13,32,55]. This reveals a double function for SUMOylation in heterochromatin repair: establishing a block to HR progression inside the heterochromatin domain and relocalizing DSBs. Furthermore, relocalization is mediated by the SUMO-targeted ubiquitin ligase (STUbL) Dgrn [32], which contains four SUMO-interacting motifs (SIMs) for binding poly-SUMOylated proteins [66], and is recruited to DSBs before relocalization [55]. STUbL and its partner, the RENi (Rad60-Esc2-Nip45) family protein dRad60 are also highly enriched at both nuclear pores and INMPs [32], suggesting a later function of these components in DSB anchoring and/or repair restart. Interestingly, Dgrn and dRad60 physically interact with the Smc5/6 complex in response to damage, suggesting that the three components establish a docking complex for repair sites at the nuclear periphery after relocalization [32] (figure 2 and table 1).

Notably, RNAi depletion of STUbL/RENi proteins, nuclear pores or INMPs affects relocalization without altering the block to HR progression inside the heterochromatin domain [32]. In the absence of these nuclear periphery components, repair sites fail to associate with the nuclear periphery and eventually return inside the domain, but Rad51 foci do not form at these sites [32]. This is different from the consequence of losing SUMOylation, which results in abnormal formation of Rad51 foci inside the heterochromatin domain [13,32,55]. These studies reveal a separation of function between the pathway that blocks HR progression and the mechanism of relocalization, with SUMOylation required for both, but STUbL and nuclear periphery components only mediating relocalization/anchoring to the nuclear envelope [13,32,55] (table 1).

What restarts repair at the nuclear periphery is still unknown, but STUbL proteins typically ubiquitinate SUMOylated targets to induce either proteasome-mediated degradation [67–71] or protein activation [72] during HR repair. Thus, ubiquitination of SUMOylated components at the nuclear periphery might remove the SUMOylated block to HR progression to restart repair (figure 2). This model predicts that the compartmentalization of SUMOylation activities inside the heterochromatin domain and of ubiquitination activities at the nuclear periphery is sufficient to regulate heterochromatin repair progression in space and time.

Notably, HR progression at the nuclear periphery also requires the presence of donor sequences, but single-strand annealing, a pathway relying on tandem repeated sequences for repair, appears surprisingly inefficient in heterochromatin [13,34,57]. This suggests that sister chromatids or homologous chromosomes relocalize together with the broken site to the nuclear periphery to provide templates for HR repair (figure 2). Accordingly, both homologous chromosomes and sister chromatids are used as templates for HR repair of *Drosophila* heterochromatin, although with a preference for the sister chromatid [57]. Homologous chromosomes are readily available as repair templates in *Drosophila* because of the characteristic mitotic pairing of the homologues in interphase [73–75] (reviewed in [76]). While the mechanisms that maintain an association between damage sites and their templates are still unknown, they likely include cohesins [77–80] and proteins required for mitotic pairing of homologous chromosomes in flies [75].

Some of the molecular details governing heterochromatin repair in mouse cells are still under investigation, but important similarities with the mechanisms discovered in *Drosophila* suggest highly conserved pathways. Similar to *Drosophila* cells, DSBs repaired via HR in mouse cells leave the heterochromatic ‘chromocentres’ before recruiting Rad51 and continuing repair [31,34,61] (figure 3). In addition, both resection and Smc5/6 are required for relocalization [34]. In both systems, RNAi depletion of Rad51 results in defective relocalization [13,34], suggesting a role of HR progression in stabilizing the positioning or anchoring of repair sites outside of the domain. However, Cas9- or ion irradiation-induced damage sites in heterochromatic satellites appear to move for a relatively short distance in mouse cells, reaching the periphery of the chromocentres before recruiting Rad51 [34,61]. Whether these sites also associate (perhaps transiently) with the nuclear periphery is unclear, and careful tracking of repair sites is required to fully understand focus dynamics in mouse cells. However, an interesting possibility is that alternative anchoring structures exist in large nuclei to limit the distance travelled and the time required for repair, along with the potential for aberrant recombination with other repeated sequences [81].

Notably, in mouse cells, relocalization of heterochromatic DSBs occurs in S/G2, but not during G1 [34], suggesting that NHEJ repair of heterochromatic DSBs does not require relocalization. This might be different in *Drosophila* tissues, where high frequency of NHEJ repair does not seem to correlate with low relocalization frequency [57], but more direct studies are necessary to establish whether NHEJ requires relocalization in *Drosophila* heterochromatin. Together, these studies revealed the importance of both relocalization and anchoring to the nuclear periphery for faithful repair of heterochromatic DSBs.

## 5. Nuclear relocalization pathways participate in repair of other repetitive sequences

Initial studies in mammalian cells detected only limited dynamics of repair sites relative to the size of the nucleus [82–90], which led to the conclusion that DSBs are substantially static in multicellular eukaryotes (reviewed in [91]). This was in striking contrast with significant movement of repair sites detected in early studies in budding yeast [67,92–96]. However, the discovery of long-range movements of repair sites for heterochromatin and other repeated sequences in *Drosophila* and mammalian cells [13,32,34,61,97,98] established a new paradigm in which extensive dynamics are also common in multicellular eukaryotes, at least for DNA repeats. This is particularly important considering that the genome of multicellular eukaryotes is largely composed of repeated sequences. Furthermore, the nuclear periphery was first identified as a preferential site for repairing relatively rare classes of ‘persistent’ DSBs, collapsed forks or telomeric lesions in yeast [67,94–96,99], while most DSBs are normally repaired in the nucleoplasm [67,96,100,101]. With the discovery of the importance of nuclear pores and INMPs in *Drosophila* heterochromatin repair [32], the nuclear periphery is emerging as an essential component for DSB repair and genome stability in multicellular eukaryotes.

Several types of nuclear dynamics have been described in the context of DSB repair, from yeast to mammalian cells (see also [102] for a recent review). First, mobilization of repair sites during inter-homologue recombination [98,100,101,103,104] likely reflects Rad51-mediated 'homology search’ (reviewed in [105]). Second, undamaged chromatin also becomes more dynamic during repair, albeit to a lesser extent than the broken site [87,101,106,107]. This could be a consequence of global chromatin relaxation [84,108] or release from nuclear anchoring structures [109–111], and might facilitate both DSB relocalization and chromatin accessibility by repair proteins. Third, fusion of repair sites into larger units, or ‘clustering’, has been observed in both euchromatin and heterochromatin [13,31,83,84,87,90], and might facilitate DSB signalling by enriching the local concentration of repair and checkpoint proteins. In human transcribed sequences, clustering potentially reflects a ‘halted’ state for HR until cells enter S phase [90]. Fourth, deprotected telomeres are mobilized in mouse cells, and this facilitates telomere fusions likely by increasing the contact probability with other chromosomes [97]. Fifth, a few chromosome territories reposition in response to damage in human fibroblasts, perhaps reflecting large-scale changes in chromatin organization [112,113]. Lastly, repair sites relocalize to specific subnuclear compartments when the lesion occurs in DNA regions that are difficult to repair such as at repeated sequences [13,31,32,34,55,93,99,114], collapsed forks [67,99], eroded telomeres [95,115], subtelomeric regions [116,117] or persistent/unrepairable DSBs (e.g. in the absence of a donor sequence for HR repair) [67,94,96,116,118–120] (figure 3). In these contexts, relocalization appears to be required to avoid aberrant recombination with ectopic repeated sequences [13,32,55,93,99] or to promote alternative repair pathways when repair is stalled [67,95,96,115,118,120] (see also [45,121–126] for recent reviews).

The relocalization pathway responsible for heterochromatin repair in *Drosophila* shares striking similarities with pathways that respond to DSBs in repeated sequences or to persistent DSBs in budding yeast (recently reviewed in [45]). For example, pioneering work in budding yeast revealed that DSBs in ribosomal DNA (rDNA) repeats leave the nucleolus before recruiting Rad51, and this requires Smc5/6 and SUMOylation by Siz2 (a dPIAS homologue) [93]. Given the abundance of tandem repeated sequences, yeast rDNA presents similar challenges to repair pathways as pericentromeric heterochromatin in multicellular eukaryotes. Further, persistent DSBs move to nuclear pores or the INMP Mps3 (a Koi and Spag4 homologue) [67,94,96,118–120]. This pathway also relies on Smc5/6, SUMOylation by Nse2 and Siz2 [67,94,96,120], and the STUbL–RENi proteins Slx5/8-Esc2 (homologues of Dgrn-dRad60). Finally, STUbL and SUMOylation are also required for targeting eroded telomeres and expanded CAG repeats to nuclear pores for HR repair in yeast [95,99,115,116].

The similarity between relocalization pathways in yeast and in *Drosophila* heterochromatin repair is particularly surprising, given that budding yeast lacks pericentromeric heterochromatin and the ‘silent’ histone marks or HP1 proteins required to relocalize heterochromatic DSBs [13]. However, the existence of similar relocalization pathways suggests that relocalization mechanisms originated early in the evolution, and have evolved to deal with the complexity of repairing the long stretches of highly repeated sequences that characterize heterochromatin in multicellular eukaryotes. One question raised by these studies is whether the repeated nature of the DNA is sufficient to trigger relocalization signals or additional levels of control (such as the presence of HP; see also next section) are required for mobilizing heterochromatic sequences.

Intriguingly, distinct SUMOylated proteins appear to generate relocalization signals in different contexts: SUMOylation of Rad52 mediates the relocalization of damaged rDNA and expanded CAG repeats [93,99]; SUMOylation of telomeric proteins and RPA (replication protein A) triggers relocalization of eroded telomeres to the nuclear periphery [115]; and H2AZ SUMOylation targets persistent DSBs to nuclear pores [94,118]. What components are SUMOylated for the spatial and temporal regulation of heterochromatin repair is still unknown, and given the abundance of SUMOylated proteins during DSB repair (e.g. [127–130]), identifying those targets is a major challenge in the field. Possible targets include histones [94,118,131], RPA [68,115,132,133], Mdc1/Mu2 [68], Smc5/6 subunits [127,129], Blm (Bloom syndrome protein) [134,135] and other repair [99,127–129] and heterochromatin [71,136] components. However, artificial targeting of poly-SUMOylated tails or the STUbL Slx5/8 is sufficient to trigger relocalization of an undamaged chromatin site to the nuclear pores in yeast [120], supporting the idea that once these targets are SUMOylated, relocalization occurs through common pathways.

Notably, not all repeated sequences move to new locations during HR repair, which would argue against a model where the presence of repeated sequences is sufficient to induce relocalization. A typical example is mouse centromeric sequences that remain associated with the minor satellite region during HR progression [34]. Further, significant differences between relocalization pathways have been identified (reviewed in [45]). In addition to specific SUMOylation targets, different pathways rely on distinct anchoring structures and repair pathways available at the nuclear periphery (reviewed in [45]). Characterizing the differences and similarities between relocalization pathways for distinct DNA sequences, and the role of both repeated sequences and silencing components in relocalization, is a necessary step to unravelling the role of nuclear architecture and dynamics in genome stability.

## 6. How do pre-existing histone marks impact heterochromatin repair?

How cells distinguish heterochromatic DSBs and channel them through a specialized repair pathway defined by distinct spatial and temporal dynamics is largely unknown. However, the unique chromatin environment in heterochromatin (figure 1*a*) likely contributes to different aspects of this response.

In *Drosophila* cells, components required for heterochromatin silencing (i.e. Su(var)3–9, SetDB1 and HP1a) are essential to block Rad51 recruitment and abnormal HR progression inside the heterochromatin domain [13] (figure 1 and table 1). Epistasis analyses place HP1a and Smc5/6 in the same pathway for blocking Rad51 recruitment [13]. HP1a also physically interacts with Smc5/6 and is required for Smc5/6 recruitment to chromatin, suggesting that the function of silencing components in blocking HR progression is, at least in part, mediated by the recruitment of Smc5/6 and its SUMO-ligase activities [13,32,55] (figure 1 and table 1). However, RNAi depletion of silencing components is not sufficient to induce Rad51 focus formation in mouse chromocentres, suggesting a more complex protection mechanism in mammalian cells.

The silent chromatin state might also enhance early repair steps by facilitating DSB signalling and/or resection in heterochromatin. Direct studies to test this hypothesis are still missing, but, intriguingly, H3K9me2/3 and HP1 proteins are transiently deposited to euchromatic DSBs [137–144], and defects in this response result in impaired DSB signalling, RPA focus formation and HR progression [139–144]. RPA typically associates with resected DSBs, suggesting that silent marks promote resection at euchromatic DSBs [139–142,144]. This is potentially mediated by the Brca1–Bard1 complex, which physically associates with HP1γ and counteracts chromatin barriers to resection [140,144–146]. The transient deposition of silent chromatin marks at euchromatic DSBs might also promote damage signalling and checkpoint activation by inducing chromatin condensation [81,143]. In agreement, inducing compaction of a chromatin array by targeting silencing components to chromatin is sufficient to trigger a DSB response in mammalian cells [81].

Whether similar activities contribute to the DSB response in heterochromatin is unclear, but the constitutive compaction and high levels of H3K9me2/3 and HP1 proteins in this domain might be sufficient to enhance DSB signalling and resection, resulting in faster progression of early HR steps. Interestingly, ATM is required to stabilize Brca1–Bard1–HP1γ associations during repair [145]. Given the importance of ATM in heterochromatin repair ([30], see also [147]), it will be important to establish whether ATM functions in this context are mediated by Brca1–Bard1–HP1γ complexes.

In conclusion, heterochromatin compaction and the unique pre-existing chromatin state could influence the initial steps of heterochromatin repair in different ways, by: (i) promoting DSB signalling and resection, thus channelling DSBs through the HR pathway, (ii) suppressing HR progression after resection, via HP1-dependent recruitment of Smc5/6 and SUMOylation and (iii) triggering relocalization, via resection activation and SUMOylation induction. Understanding the impact of silencing on resection and relocalization of heterochromatic DSBs, and mechanisms available in mammalian cells to regulate these repair steps, are important goals for future studies.

## 7. What is the role of chromatin expansion in heterochromatin repair?

In the absence of damage, the heterochromatin domain appears compact and shows very limited dynamics [13,20]. However, DSB formation results in a striking expansion of the heterochromatin domain in *Drosophila* and mouse cells [13,34,55,60,148] (figure 3). In *Drosophila* cells, expansion starts minutes after DSB formation by IR, and peaks during relocalization of repair sites resulting in up to approximately 50% more volume occupied in the nucleus [13,55]. This response is also associated with the formation of dynamic protrusions of the heterochromatin domain, and is followed by partial contraction [13]. In fly cells, the mechanisms responsible for expansion include checkpoint kinases, particularly ATR, and resection components. Interestingly, the same components are required for DSB signalling and relocalization of heterochromatic DSBs [13] (figure 3), suggesting that expansion facilitates early steps of repair and/or the mobilization of repair sites in flies. The nature of the chromatin changes leading to expansion is still unclear, as is the impact of expansion on relocalization, but this response does not correlate with a spreading of HP1a along the chromosomes in *Drosophila* [13], and it more likely reflects a general relaxation of the heterochromatin domain.

Notably, global chromatin relaxation followed by contraction in response to damage does not appear to be unique to the heterochromatin domain, given that similar phenomena were described in studies examining the chromatin behaviour in the entire nucleus [84,108,148]. For example, in human cells global DNA access to digestion with micrococcal nuclease (MNase) increases in response to IR [108], and this response is dependent on Kap1 Ser824 phosphorylation by ATM [108]. Further, damage-induced release of the histone H1 from chromatin promotes global chromatin relaxation in mouse ES cells and in yeast, facilitating DSB signalling and resection [149]. More recently, studies in yeast revealed that chromatin remodellers and checkpoint-induced degradation of histone proteins promote global chromatin dynamics during HR repair, which might relate to a more ‘accessible’ chromatin state [150,151]. While global chromatin relaxation is frequently observed in response to damage, this response might be particularly important in contexts where large-scale nuclear motions are critical elements of the repair response, and where the chromatin is potentially less accessible or less dynamic, such as in heterochromatin. In agreement with this idea, blocking ATM or Kap1 S824 phosphorylation has a stronger effect on repair in heterochromatin than in the rest of the genome in mouse cells [30]. ATM is also required for heterochromatin expansion in *Drosophila* [13], but it is still unclear whether Kap1/Bonus and its phosphorylation also contribute to this response.

Finally, while heterochromatin expansion might facilitate the movement of repair sites, such as by releasing constraints due to compaction and by facilitating the ‘looping’ of DNA sequences to outside the domain for repair, this global response is not sufficient for relocalization of DSBs [55]. In fact, relocalization defects have been observed even in conditions when expansion is normal (e.g. after Nse2/Qjt RNAi in *Drosophila* cells) [55], genetically separating heterochromatin expansion from relocalization (table 1). Furthermore, blocking chromatin relaxation in mouse cells (e.g. by Kap1pS824 mutation) does not impair relocalization of DSBs but it affects heterochromatin repair [34], suggesting a later function of relaxation in DSB processing (see also next section). This is consistent with studies showing that artificial induction of silencing and compaction of a chromatin locus does not affect early damage signalling but it impairs repair progression [148]. Thus, more studies are required to understand the mechanisms of heterochromatin expansion, the chromatin changes involved, and the significance of expansion to repair progression, but this response likely facilitates heterochromatin repair by positively contributing to the accessibility and dynamics of this domain.

## 8. How do local chromatin changes contribute to heterochromatin repair?

In addition to global reorganization of the heterochromatin domain (i.e. expansion and contraction), several studies suggest that local chromatin changes (i.e., changes proximal to the DSB site) also participate in early and late steps of heterochromatin repair (figure 3). The specifics of this response still need to be understood, but a general view is that chromatin transitions to a more accessible state to facilitate repair progression, by nucleosome repositioning, chromatin relaxation or histone modification changes (e.g. via release of ‘silent’ histone marks, or the acquisition of active marks; see also [147]). Here, we will discuss evidence suggesting that HP1 proteins and the HP1-interactor Kap1 are key targets of this regulation, as their local release or modification promotes nucleosome reorganization and/or chromatin relaxation, and those responses facilitate early and late steps of heterochromatin repair. We will also point to specific chromatin modifiers potentially involved this response.

HP1 proteins are directly targeted by post-translational modifications during heterochromatin repair. In mouse cells, laser-induced DSBs in heterochromatin result in HP1β phosphorylation by CK2 (casein kinase 2) and HP1β release from the chromatin, which might promote chromatin opening at repair sites [60] (figure 3). Blocking this pathway severely affects H2AX phosphorylation, revealing its importance in the initial steps of DSB signalling [60].

Local HP1 release from the chromatin might also be required at later repair steps, to promote HR progression after relocalization. In *Drosophila* cells, Rad51 recruitment to heterochromatic DSBs correlates with a significant reduction of HP1a signals at repair sites [13]. Similarly, induction of DSBs in heterochromatin by laser radiation leads to the loss of HP1a at Rad51-containing repair foci [13], suggesting that HP1a is locally ‘loosened’ at or displaced from heterochromatic DSBs during repair progression (figure 3). Given the mutual exclusion between HP1a and Rad51, it has been proposed that HP1a removal is necessary for Rad51 recruitment [13]. In agreement, RNAi depletion of HP1a results in abnormal Rad51 recruitment inside the heterochromatin domain [13]. These observations lead to a model that identifies two critical roles of HP1a in the spatial and temporal regulation of heterochromatin repair in flies: (i) HP1a presence at early steps of repair is needed to recruit Smc5/6 and block HR progression and (ii) the local displacement of HP1a after relocalization of repair sites to the nuclear periphery might be required to enable Rad51 recruitment and repair progression (figure 3). More studies are needed to understand the extent to which HP1a is released at heterochromatic DSBs and how these changes impact specific repair steps.

In addition to HP1, the chromatin component Kap1 is targeted by several post-translational modifications in response to DSBs, which earmarks this component as a central regulator of chromatin dynamics during repair (reviewed in [152,153]). For example, Kap1 S824 phosphorylation is enriched at repair foci before spreading to the rest of the nucleus in mouse cells, suggesting a local function at DSBs [108,136,154,155]. Accordingly, Kap1 pS824 promotes local release of the chromatin remodeller Chd3 from the chromatin proximal to DSBs, likely resulting in local chromatin loosening [136,156] (figure 3). Similarly, Kap1 S473 phosphorylation by Chk2 [156] has been reported to weaken Kap1–HP1β interaction and increase HP1β mobilization in response to damage [157], potentially contributing to local and/or global heterochromatin relaxation during repair. Intriguingly, STUbL-dependent degradation of SUMOylated Kap1 pS824 also facilitates HR repair in mammalian cells [71], raising the possibility that Kap1 may be targeted by the proteasome to promote HR progression after DSB relocalization to outside the heterochromatin domain [153].

HP1β mobilization and Kap1 phosphorylation have also been observed at DSBs in euchromatin [60,108,158], but similar to global chromatin relaxation, these responses might be particularly important to promote chromatin accessibility in compact heterochromatic regions during repair. Accordingly, HP1 removal, constitutive phosphorylation of Kap1 or Chd3 loss, alleviates the ATM requirement for DSB repair specifically in heterochromatic regions in mouse cells [30,136].

In addition to chromatin reorganization resulting from HP1 and Kap1 phosphorylation, changes in histone modifications are likely to contribute to early and late steps of HR repair in heterochromatin. Direct studies addressing the role of chromatin modifiers in heterochromatic DSB repair are still missing, but candidates for these functions include the histone acetyltransferases Tip60, p300, the histone demethylase Kdm4B, and the chromatin remodellers SWI/SNF and ISWI (for an overview of the roles of these components in DSB repair, see also [159–163]). Tip60 directly associates with H3K9me3 through its chromodomain, and this association is essential for Tip60 ability to induce histone acetylation, chromatin relaxation and HR repair [142,164,165]. Given the abundance of H3K9me3 in heterochromatin, a specific role of Tip60 in this domain has been previously suggested [165]. In addition to Tip60, p300 becomes enriched at HP1α-containing chromatin in response to UV irradiation, suggesting a role for p300 in heterochromatin repair [166]. p300 promotes chromatin relaxation and HR repair via H3/H4 acetylation and the recruitment of the chromatin remodelling complex SWI/SNF in euchromatin [167,168]. Whether these responses facilitate heterochromatin repair still awaits investigation. Intriguingly, yeast SWI/SNF is required for strand invasion of silenced chromatin in biochemical assays [169], suggesting a role for this complex in chromatin accessibility of heterochromatic donor sequences for the progression of HR repair. Further, *Drosophila* Kdm4B is recruited to heterochromatin to reduce H3K9me3 levels in response to UV damage [170], and human Kdm4B associates with DSBs to promote repair [171], pointing to a potential role for this histone demethylase in DSB repair of heterochromatic regions. Finally, the ISWI chromatin remodeller has been involved in nucleosome repositioning after Chd3 dispersal during heterochromatin repair [172].

Together, these studies support a model where complex chromatin dynamics, including chromatin loosening and/or nucleosome repositioning, participate in heterochromatic DSB repair. However, more studies are needed to establish the specific function of chromatin remodellers, histone modifiers, or Kap1 and HP1 modifications, in these chromatin changes. More work is also needed to understand how chromatin dynamics impact early and late steps of heterochromatin repair, including DSB signalling, repair pathway choice, chromatin looping to outside the domain, relocalization of DSBs and HR repair progression.

## 9. Conclusion and perspectives

Significant efforts in the past decade have begun shedding light on the mysterious mechanisms responding to DSBs in heterochromatin, revealing several unexpected and unique features of repair pathways in this domain. These discoveries challenged the previous view that DSBs are mostly static in the nucleus of multicellular eukaryotes, revealing striking dynamics of both the heterochromatin domain and repair sites. Further, counterintuitive to what would be a ‘safe’ repair strategy, heterochromatin is preferentially repaired by HR in S/G2 cells. To mitigate the risks of HR with ectopic sequences on other chromosomes, DSBs relocalize to outside of the domain and even associate with the nuclear periphery before strand invasion. In *Drosophila* cells, the coordination of repair progression with nuclear dynamics includes several key steps: (i) DSBs are quickly processed for HR repair inside the heterochromatin domain while Rad51 recruitment is temporarily blocked by SUMOylation, (ii) resection and checkpoint activation trigger DSB relocation to the nuclear periphery and (iii) signalling mechanisms at the nuclear periphery enable repair restart in a ‘safe’ environment. Despite significant progress in this field, many questions remain unanswered. For example, the targets of SUMOylation are still unknown and the specific effects of SUMOylation and ubiquitination on these components have not been investigated. The mechanism of relocation to the nuclear periphery is also unclear. Specifically, it is not known if Brownian/sub-diffusive motion followed by nuclear periphery anchoring is sufficient to induce relocalization, or if active forces are involved. The nature and function of local and global chromatin responses in these nuclear dynamics are also largely unknown. Finally, major efforts started unravelling the similarities between *Drosophila* and mammalian cells, providing exciting new insights. Given that HR [173–176], heterochromatin silencing [177,178] and the nuclear periphery [179] are typically deregulated in cancer cells and become progressively dysfunctional with ageing [180–183] (see also [45] for a recent review), understanding heterochromatin repair mechanisms is expected to open new avenues for the treatment of cancer and other ageing-dependent human diseases. The tools are now in place for exciting new discoveries in this field in the near future.

## Data accessibility

This article has no additional data.
